# Cross-Modal Sentiment Sensing with Visual-Augmented Representation and Diverse Decision Fusion

**DOI:** 10.3390/s22010074

**Published:** 2021-12-23

**Authors:** Sun Zhang, Bo Li, Chunyong Yin

**Affiliations:** School of Computer and Software, Nanjing University of Information Science & Technology, Nanjing 210044, China; szhang23@sina.com (S.Z.); lbo923@yeah.net (B.L.)

**Keywords:** decision fusion, multimodal learning, representation fusion, social network

## Abstract

The rising use of online media has changed the social customs of the public. Users have become accustomed to sharing daily experiences and publishing personal opinions on social networks. Social data carrying emotion and attitude has provided significant decision support for numerous tasks in sentiment analysis. Conventional methods for sentiment classification only concern textual modality and are vulnerable to the multimodal scenario, while common multimodal approaches only focus on the interactive relationship among modalities without considering unique intra-modal information. A hybrid fusion network is proposed in this paper to capture both inter-modal and intra-modal features. Firstly, in the stage of representation fusion, a multi-head visual attention is proposed to extract accurate semantic and sentimental information from textual contents, with the guidance of visual features. Then, multiple base classifiers are trained to learn independent and diverse discriminative information from different modal representations in the stage of decision fusion. The final decision is determined based on fusing the decision supports from base classifiers via a decision fusion method. To improve the generalization of our hybrid fusion network, a similarity loss is employed to inject decision diversity into the whole model. Empiric results on five multimodal datasets have demonstrated that the proposed model achieves higher accuracy and better generalization capacity for multimodal sentiment analysis.

## 1. Introduction

Social media has become the dominant approach to sharing daily experiences and publishing individual opinions, and is a benefit from the rapid development of mobile devices and communication technologies [1]. Personal sentiments are contained in online user-generated content, which have a direct relevance to users’ behaviors in offline lives. Sentiment analysis is a significant technology that builds the bridge between user-generated data and potential sentiment, which can provide decision support for massive applications. For example, a product review on an e-commerce platform could contain the real demand and interest point of the customer, which will help the manufacturers to promote product quality. For investors, the emotions of shareholders are exploited to predict the market trend and avoid investment risks. For the government, a social platform is an important approach to collect public opinions that are further employed for policy making and evaluation.

Conventional methods for sentiment analysis only focus on textual contents and learning representations for different structures, e.g., word, phrase, sentence, and document. However, the composition of user-generated content has been more complex and diverse in recent years. The plain textual description is gradually replaced by the mixture of images and texts [2]. Any source or form of information can be considered as a type of modality, and a social network is such a complex environment full of multiple modalities, where text and image are two of the most dominant modalities. Multimodal user-generated contents have brought new challenges to various tasks of sentiment analysis. Firstly, the format and structure of image and text are heterogeneous. It requires different methods to process and extract discriminative features. Secondly, the model for multimodal sentiment classification should explore the interaction relationship and relevant features between modalities. Finally, Verma et al. [3] pointed out that the individual modality has its unique intra-modal characteristics. It is necessary to capture both the common inter-modal and unique intra-modal information.

Existing methods for multimodal sentiment classification are grouped into three categories according to the fusion stage, i.e., early data fusion, intermediate representation fusion, and late decision fusion [4]. Early data fusion focuses on integrating information from multiple data sources or views into one feature vector, which contains redundant noises and cannot completely capture the relationship among modalities. The intermediate representation fusion can extract individual characteristics from each modality and fuse them into a joint representation. It concerns mining common inter-modal information and achieving a higher accuracy with the complementary effect between the modalities. Multimodal information is aggregated in the decision-making stage for late decision fusion, and the final decision is determined by integrating the predictions from independent models that are trained with only single-modal information. The late decision fusion has better robustness and generalization by capturing the unique intra-modal information, whose central idea is similar to ensemble learning.

Intermediate representation fusion is the most common method in multimodal sentiment analysis. Whether directly concatenating [5,6] or generating joint representations by attention mechanism, most researches assume that there is a one-to-one correspondence within the text-image pair [7]. You et al. [8] pointed out that different modalities are consistent for expressing the same sentiment and a consistency constraint was added to implicitly enforce the similarity between prediction functions of each modality. In the follow-up study, they proposed a tree-structured model [9] to explicitly align textual words and visual regions for learning joint representations.

**Figure 1:**
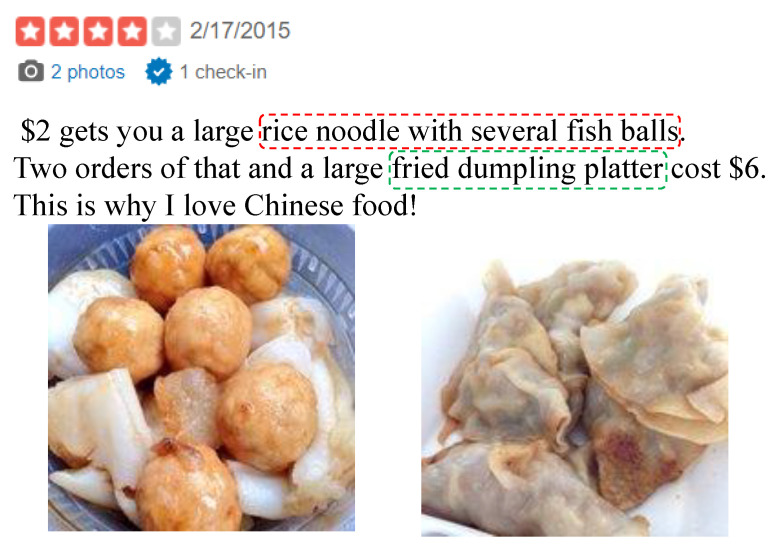
An image within a review tends to focus only on one thing that tends to be mentioned in the textual content, while the sentences within a review tend to involve several things and sentiment-bearing words.

However, for blog posts and product reviews, multiple images are attached with the textual content to enhance the vividness and credibility of description. The correspondence between text and images are unbalanced. Truong et al. [10] pointed out that images only play an augmentative role in product reviews, rather than an independent role, which means images are unable to deliver complete information on their own. For example, the restaurant review shown in Figure 1 has two types of modal contents, including two images and several sentences describing foods. According to the example, we can observe that an image within a review tends to focus only on one thing that tends to be mentioned in the textual content, while the sentences within a review tend to involve several things and sentiment-bearing words. Therefore, an image can help identify the important parts of the textual review, but the cues of sentiment polarity provided by an image are rare.

Given the pair (T,G) of textual and visual contents, *T* is a sequence of *L* words $\left\{w_{1},w_{2},\ldots ,w_{L}\right\}$ and *G* is a set of *N* images $\left\{a_{1},a_{2},\ldots ,a_{N}\right\}$. Our research objective is to learn the mapping function between (T,G) and sentiment label $y\in R^{C}$. A hybrid fusion network (HFN), which integrates representation and decision fusion, is proposed in the paper to capture the interactive inter-modal and unique intra-modal information for better performance in sentiment classification. In the stage of representation fusion, the fine-tuned BERT [11] is utilized to extract the embedding representations of words while the pre-trained VGG16 [12] is employed for visual representations. Following the idea of VistaNet, a multi-head visual attention is proposed to fuse multimodal representations, in which multiple images are utilized as queries to locate and measure the importance of words. For a better generalization capability and to capture unique intra-modal information, a decision fusion method is proposed to ensemble prediction results from multiple independent classifiers. The main contributions of this work are three-fold:

- A hybrid fusion network is proposed to capture the common inter-modal and unique intra-modal information for multimodal sentiment analysis;
- A multi-head visual attention is proposed for representation fusion to learn a joint representation of visual and textual features, in which the textual content provides the principal sentiment information and multiple images are employed as an augmentative role;
- A decision fusion method is proposed in the late fusion stage to ensemble independent prediction results from multiple individual classifiers. The cosine similarity loss is exploited to inject decision diversity into the whole model, which has been proven to improve generalization and robustness.

## 2. Related Work

### 2.1. Sentiment Analysis

Sentiment analysis is a significant technology that builds the bridge between user-generated data and potential sentiment, which have massive applications in diverse fields. For markets, understanding the feelings and preferences of customers can contribute to personalized recommendations and marketing [13]. For individuals, recognizing and monitoring the personal psychology states are crucial for mental health and emotion management [14]. Conventional methods about sentiment analysis are based on representation learning to capture semantic and sentimental information. Text sentiment analysis first appeared in mid-1990s, which has several sub-tasks including opinion mining, emotion mining, and polarity classification [15]. Despite the different terms, the research objectives are similar, which is to detect and classify the feelings and attitudes about specific events or objects. Early studies of text sentiment analysis focus on the extraction of sentiments from semantic lexicons by building sentiment dictionary and matching specific words [16]. It is tedious and time-consuming to build the dictionary which also neglects the contextual information. With the development of deep learning and text classification, loading and fine-tuning the pre-trained language models have become a popular approach to obtaining the embedding representations of texts [17,18,19]. Then, convolution-based neural networks [20,21], recurrent-based neural networks [22,23], or attention mechanism-based models [24,25] could be employed to learn high-level semantic features. Finally, a task-specific network is constructed to predict sentiment labels for downstream applications.

For uni-modal sentiment analysis, textual features are considered to have a better capacity of sentiment expression, because words can carry more or less information relevant with sentiments and attitudes [26]. The methods of text sentiment analysis have been continuously refined and improved with the development of text classification techniques. Both text classification and text sentiment analysis require the extraction of semantic information which makes them technically similar.

Image sentiment analysis has received less attention than text sentiment analysis, although there is great progress on image classification tasks. Image sentiment analysis has an essential difference from image classification, since it needs high-level abstraction for semantic understanding, rather than the low-level visual features extracted by classification models [27]. Machajdik et al. [28] proposed that the core of image sentiment analysis is efficiently learning representations from the low-level visual and high-level semantic information. In addition, there is an implicit relationship between visual sentiment and human knowledge background which makes the same image bring a different emotional experience to different observers.

Borth et al. [29] introduced the psychology theories and constructed more than 3000 adjective-noun pairs with sentiment labels. Then, the mid-level semantic features could be extracted from images by matching with recorded adjective-noun pairs. You et al. [30] had an implementation of domain transferring from Twitter to Flickr for binary sentiment classification. A convolution neural network was trained to recognize sentiment information of different local visual regions by Yang et al. [31] and attention scores were assigned to each local region for obtaining high-level representation. Guillaumin et al. [32] found that the additional textual information corresponding to the images are helpful for understanding visual contents and achieving a higher accuracy of image classification, which also make communities pay more attention to multimodal fusion.

### 2.2. Multimodal Sentiment Analysis

Multimodal learning can attach the information of relevant modalities to textual contents, which provides evidence from different views to understand the semantic and sentiment. Baltrusaitis et al. [1] summarized the challenges and problems of multimodal learning. There are four aspects related to multimodal sentiment analysis:

- Representation. The first challenge of multimodal learning is how to extract discriminative features from heterogeneous multimodal data. Texts are usually denoted by discrete tokens, while images and speeches are composed of digital and analog signals. The corresponding methods of feature extraction are required for different modalities to learn effective representations.
- Transformation. The second challenge is learning the mapping and transformation relationship among the modalities, which can eliminate the problems of missing modality, and discover the correlation between the modalities. For example, Ngiam et al. [33] proposed to learn a shared representation between modalities with restricted Boltzmann machine in an unsupervised transformation manner.
- Alignment. The third challenge is to correctly explore the direct corresponding relationship between different modal elements. Truong et al. [10] employed the alignment relationship between visual and textual elements to locate the contents relevant with opinions and attitudes. Adeel et al. [34] utilized the visual features to eliminate the noises in the speech based on the consistency between audio and visual signals.
- Fusion. The forth challenge is to integrate and refine the information from different modalities. The contributions of each modality to different tasks are variant. The fusion of features is a process of removing noises and extracting relevant information.

Most researches about multimodal sentiment analysis have focused on the feature fusion to construct joint representation. Early data fusion-based methods focus on the fusion of multi-view or multi-source information. Perez et al. [35] extracted features from visual, textual, and acoustic views to recognize the utterance-level sentiment for video data. Poria et al. [36] firstly employed the convolution neural network to learn the representations of image and text, then several classifiers based on kernel learning were employed to fuse multi-view features. The early fusion only concerns each modality separately without exploring interactive information, which neglects the complementary information among the modalities.

**Figure 2:**
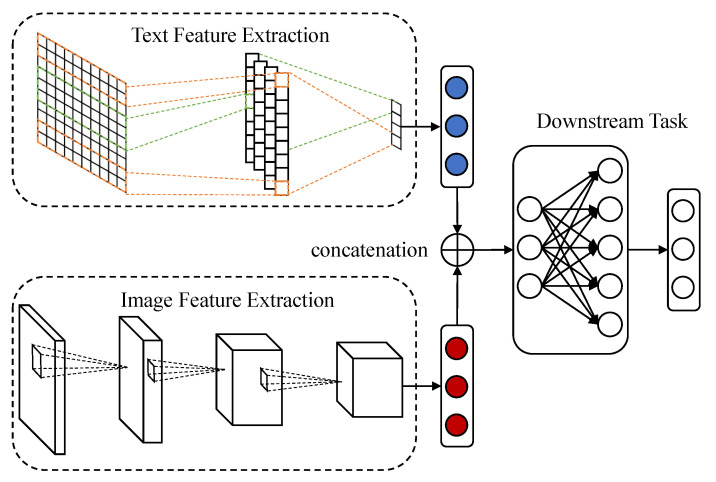
A simple and common method for representation fusion is to concatenate feature vectors extracted by different pre-trained networks.

The intermediate representation fusion aims to capture the relationship between the modalities for learning more discriminative representations. A simple and common method of representation fusion is to directly concatenate features that are extracted by neural networks with various architectures or pre-trained models as shown in Figure 2. Gogate et al. applied 3D-CNN to extract features from different modalities and concatenated them into the vector representation for emotion recognition [37] and deception detection [38]. Hu et al. [5] utilized the pre-trained Inception to extract visual features and GloVe to encode textual contents, while Chen et al. [6] employed AlexNet and Word2Vec for feature extraction. Zadeh et al. [39] pioneered Tensor Fusion Network (TFN) for multimodal sentiment analysis and the outer-product of two feature vectors is considered as the fusion result. TFN could provide both bi-modal and uni-modal information, but the dimension of outer-product tensors exponentially increases with the number of modalities which makes it unscalable. To alleviate the problem of scalability, Liu et al. [40] proposed Low-rank Multimodal Fusion (LMF) to approximate the result of an outer-product.

The attention mechanism is a better approach to aggregating the contextual information and capturing the interactive relationship between the modalities. The bidirectional attention between image and text was conducted after extracting global and local information from images in [41]. Yu et al. [42] extracted visual and textual features respectively by the pre-trained ResNet-512 and BERT. Then, the joint representation was generated by the multi-head attention. A multimodal transformer was extended to a sequential multimodal problem by Tsai et al. [43], which was able to be directly applied to unaligned sequences. Similarly, the methods based on the architecture of the transformer or multi-head attention were exploited in different fields, such as the cross-modal dialogue system [44] and video retrieval [45]. The excellent performances have demonstrated the effectiveness of multi-head attention for the cross-modal fusion. A gated mechanism could be considered as a special variant of attention mechanism, which also be employed for the cross-modal fusion. Kumar et al. [46] proposed a conditional gated mechanism to modulate the information during mining inter-modal interaction.

The late fusion is implemented in the decision stage and its idea is similar to ensemble learning which can capture the unique intra-modal information and improve the generalization capability. There are also several alternative approaches for decision fusion. Verma et al. [3] trained a neural network to learn the weight coefficients after concatenating different decisions, while Huang et al. [4] controlled the contribution to the final decision of text, image, and fusion representations by empiric hyper parameters. A special type of visual-textual data was investigated by Liu et al. [47]. Graphics Interchange Format (GIF) has received huge popularity in social networks and users usually publish animated GIFs with short textual contents to express individual emotions and sentiments. Sentiment prediction scores from visual and textual parts were weighted in the late fusion stage. Since the neural network could fit arbitrary functions in theory, it is more robust to train a neural network for making the final decision, which also reduces the number of hyper parameters.

## 3. Hybrid Fusion Network

The methods about multi-head visual attention and decision fusion are detailed in this section. Our research is oriented to social applications in which user-generated content consists of a textual paragraph and multiple images. Given the pair (T,G) of textual and visual contents, *T* is a sequence of *L* words $\left\{w_{1},w_{2},\ldots ,w_{L}\right\}$ and *G* is a set of *N* images $\left\{a_{1},a_{2},\ldots ,a_{N}\right\}$. The objective is to learn the mapping function between (T,G) and sentiment label $y\in R^{C}$.

Most existing methods of multimodal sentiment analysis usually concern capturing the inter-modal relationship, fusing the representations and making the prediction based on the complementarity between modalities. However, as mentioned in [3], each modality has their own unique characteristics and the expressed sentiments are different. Therefore, a hybrid fusion network, consisting of the intermediate representation fusion and the late decision fusion is proposed to capture both the inter-modal and intra-modal information for multimodal sentiment classification.

**Figure 3:**
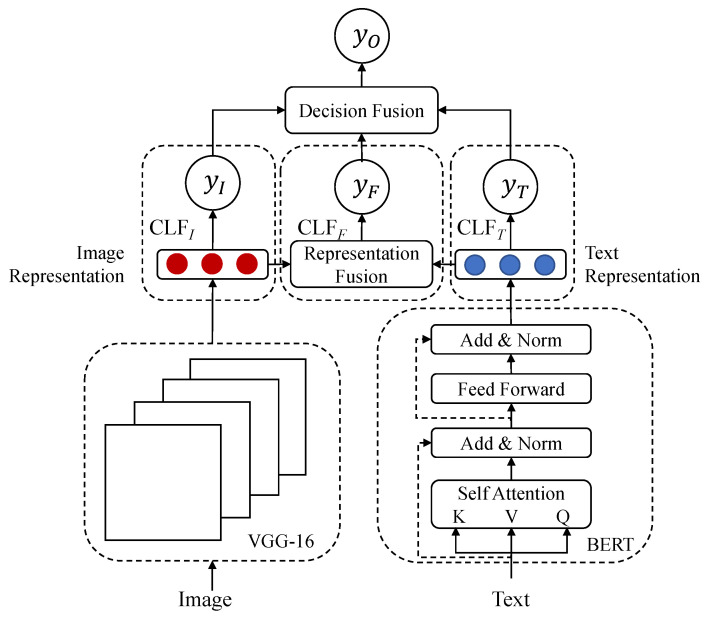
HFN (Hybrid Fusion Network) consists of two feature extractors, three individual classifiers, a representation fusion module, and a decision fusion module.

As shown in Figure 3, HFN is composed of the text feature extractor, image feature extractor, representation fusion module, decision fusion module, and three individual classifiers. Our research is mainly conducted on the multimodal dataset proposed in VistaNet, which only provides extracted visual features, rather than original images. Therefore, the 4096-dimensional feature vector is employed as the image representation $VGG16\left(G\right)=\left\{g_{i}|g_{i}\in R^{4096},i=1,2,\ldots ,N\right\}$ which is output from the last fully connected layer of VGG16. We fine-tune the pre-trained BERT as the text feature extractor, and each word is encoded as an embedding vector $\mathrm{BERT}\left(T\right)=\left\{T_{i}|T_{i}\in R^{d=768},i=1,2,\ldots ,L\right\}$.

### 3.1. Visual and Textual Representation Fusion

**Figure 4:**
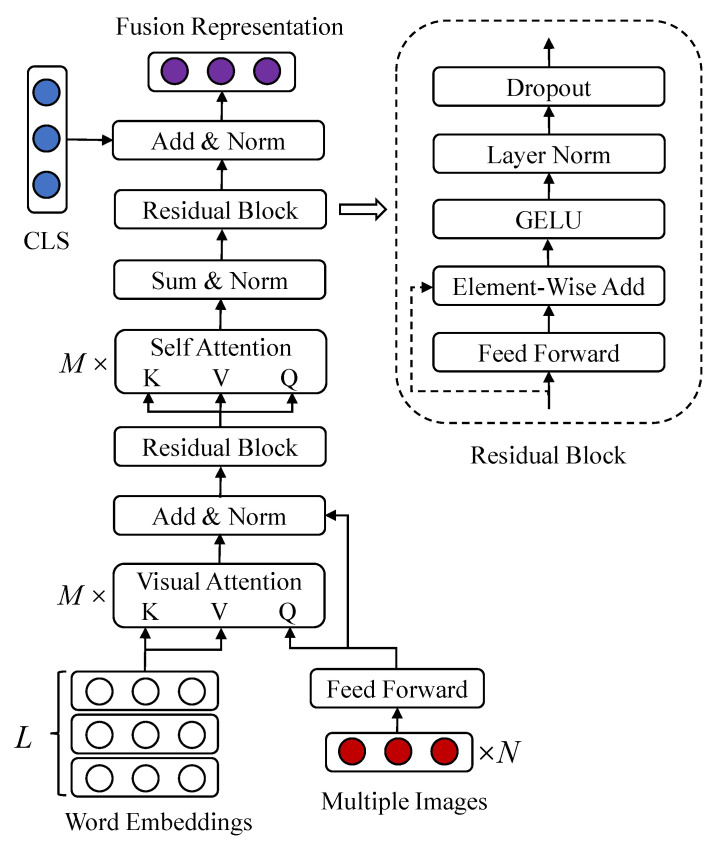
The visual features of *N* images are utilized as queries, while *L* word representations are employed as keys and values in multi-head visual attention.

The representation fusion is a crucial issue in our hybrid fusion network. Most attention-based fusion methods are bidirectional which aim to align the textual entity with the visual region. However, for online blogs and product reviews, sentiment information expressed by the textual content is the principal part, while the visual content only enhances the vividness of textual content. Therefore, the visual representation is only utilized to measure the importance of different words in textual content. Different from the dot-product visual attention in VistaNet, a multi-head visual attention is proposed for representation fusion. As shown in Figure 4, the representations of multiple images $G=\left\{g_{1},g_{2},\ldots ,g_{N}\right\}\in R^{4096\times N}$ are exploited as the queries, and the embedding vectors of words $T=\left\{T_{1},T_{2},\ldots ,T_{L}\right\}\in R^{d\times L}$ are utilized as the keys and values. Firstly, image representations are mapped into the vector space with the same dimension as text representations by a fully connected layer $I=W_{g}G+b_{g}\in R^{d\times N}$. The weighted result of each attention head is calculated as:

(1) $${\mathrm{Head}}_{i}\left(I,T\right)=\mathrm{softmax}\left(\frac{{\left(W_{i}^{Q}I\right)}^{T}\left(W_{i}^{K}T\right)}{\sqrt{d/M}}\right){\left(W_{i}^{V}T\right)}^{T}$$

where $\left\{W_{i}^{Q},W_{i}^{K},W_{i}^{V}\right\}$ are different learnable parameter matrices for linearly projecting query, key, and value into $\sqrt{d/M}$-dimensional vector spaces. *M* denotes the number of attention heads which is a hyper parameter. Since multiple images are corresponded to one textual paragraph in our research problem, the shape of weighted result from a single attention head is $N\times \sqrt{d/M}$. Then, the final weighted result ${\mathrm{MATT}}_{I,T}\in R^{N\times d}$ could be obtained after concatenating the results from *M* attention heads, and the operation of concatenating is denoted as Concat in Equation (2):

(2) $${\mathrm{MATT}}_{I,T}=\mathrm{Concat}\left[{\mathrm{Head}}_{1},{\mathrm{Head}}_{2},\ldots ,{\mathrm{Head}}_{M}\right].$$

Similar to the multi-head self-attention in BERT, a residual connection followed by layer normalization (LN) is placed between the query vector and next layer. LN could prevent the gradient explosion caused by large accumulated gradients, and residual connection could alleviate the gradient vanishment occurred in back propagation. In HFN, each residual block (denoted as Res) is composed of a fully connected layer, element-wise additive, Gaussian Error Linear Units (GELU), LN, dropout, and shortcut connection.

(3) $$Z=\mathrm{Res}\left(\mathrm{LN}\left({\mathrm{MATT}}_{I,T}^{T}+I\right)\right)\in R^{d\times N}.$$

The original visual information and related textual information of images are captured by intermediate features $Z\in R^{d\times N}$ now. Considering the relevance of visual content, there are duplication and potential correlation between query results which are delivered to intermediate features. Therefore, a multi-head self-attention is employed to refine and fuse the information of intermediate features in Equation (4):

(4) $${\mathrm{Head}}_{i}\left(Z\right)=\mathrm{softmax}\left(\frac{{\left(W_{i}^{Q}Z\right)}^{T}\left(W_{i}^{K}Z\right)}{\sqrt{d/M}}\right){\left(W_{i}^{V}Z\right)}^{T}.$$

The multi-head self-attention is similar to Equation (1), while the input matrix is utilized as query, key, and value at the same time. After the results of each attention head are concatenated into a vector ${\mathrm{MATT}}_{Z}\in R^{d\times N}$ as the same operation of Equation (2), element-wise additive along *N*-dimension and LN are utilized to integrate the fusing information corresponding to multiple images:

(5) $$Z^{\prime }=\mathrm{Res}\left(\mathrm{LN}\left(\mathrm{sum}\left({\mathrm{MATT}}_{Z}^{T}\right)\right)\right)\in R^{d}.$$

For most methods of text sentiment analysis, $T_{\mathrm{CLS}}$, the embedding representation of token [CLS], is usually considered as the sentence-level or document-level feature after fine-tuning BERT. $Z^{\prime }$ is the attention weighted result on word embedding representations and it has the same level information with $T_{\mathrm{CLS}}$. Therefore, the element-wise addictive result of $Z^{\prime }$ and $T_{\mathrm{CLS}}$ is directly employed as the fusion representation *F* after layer normalizing:

(6) $$F=\mathrm{LN}\left(Z^{\prime }+T_{\mathrm{CLS}}\right)\in R^{d}.$$

### 3.2. Decision Fusion and Injecting Diversity

Considering the unique intra-modal information, individual classifiers ${\mathrm{CLF}}_{F},{\mathrm{CLF}}_{T},$, and ${\mathrm{CLF}}_{I}$ are respectively trained with different representations to generate diverse decision supports. ${\mathrm{CLF}}_{F}$ denotes the classifier trained with the fusion representation *F*. ${\mathrm{CLF}}_{T}$ is the classifier expected to learn the decision space with only textual representation. Similarly, ${\mathrm{CLF}}_{I}$ denotes the classifier trained with only visual representations. To prevent the over-fitting in downstream classification task, only one fully connected layer is employed as the classifier to learn the mapping from high-level representation to target label. The embedding representation of the token [CLS], usually considered as the document-level feature of textual content, is input into ${\mathrm{CLF}}_{T}$ for learning the decision $y_{T}\in R^{C}$. The max-pooling result over multiple image representations $G\in R^{4096\times N}$ is utilized as the input of ${\mathrm{CLF}}_{I}$ to make independent decision $y_{I}\in R^{C}$. Similarly, the decision $y_{F}\in R^{C}$ is generated from ${\mathrm{CLF}}_{F}$ based on the fusion representation *F*. Then, a neural network is trained to measure the confidence of concatenated decisions $y_{C}=\mathrm{Concat}\left[y_{F},y_{T},y_{I}\right]\in R^{C\times 3}$ output from three classifiers, and final decision $y_{O}\in R^{C}$ could be determined by attention fusion as:

(7) $$y_{O}=\mathrm{softmax}\left(W_{C}y_{C}+b_{C}\right)y_{C}^{T}.$$

For most methods about multimodal sentiment analysis [3,4], the cross entropy between prediction result $y_{O}$ and true label *y* is employed as the loss function for model training. It expects the final decision could be closer to true labels without considering the accuracy of individual classifiers before the decision fusion. We expect the independent classifiers could also output accurate predictions, rather than further extract features. Therefore, the cross entropy loss values of individual classifiers are also considered to be a part of decision loss as:

(8) $$\mathrm{decision}\_\mathrm{loss}=\sum_{i}-y\log y_{i};i\in \left\{O,F,T,I\right\}.$$

In addition, the late decision fusion aims to improve generalization with the integrated decisions. If three classifiers always make the same decisions, the decision fusion module will degenerate into a linear additive function. Therefore, the cosine similarly is utilized as a penalty term for decision diversity as follows:

(9) $$\mathrm{similarity}\_\mathrm{loss}=\sum_{i}\sum_{j\neq i}\cos\left(y_{i},y_{j}\right);i,j\in \left\{F,T,I\right\}$$

where $\cos\left(a,b\right)=\frac{a\cdot b}{{∥a∥}_{2}\times {∥b∥}_{2}}$ denotes the cosine similarity. Finally, the loss function utilized in our training process is:

(10) loss=decision_loss+α×similarity_loss.

The hyper parameter α is exploited to balance the influence of cosine similarity penalty in the training process. It should not be too large, because the decision diversity is expected to be optimized after the classifier is relatively convergent and larger α could reduce the accuracy of individual classifiers, even for the entire framework.

## 4. Experiments and Analysis

Comprehensive experiments are conducted to evaluate the validity of multi-head visual attention and the decision fusion method in this section. All the codes are written in Python 3.6.9 on Ubuntu 18.04 and the framework of deep learning is PyTorch 1.4.0. Intel Core i9-9900K CPU@3.6 GHz ×16 and GeForce RTX 2080 GPU are utilized to accelerate the training process. Due to the limitation of graphics memory, the fine-tuning process of BERT was completed on Google Colab, which provides NVIDIA Tesla K80 GPU for researchers.

### 4.1. Comparative Experiments on Multimodal Yelp Dataset

Five datasets from three social platforms are employed to evaluate our proposed model. The first dataset is published with the baseline model (i.e., VistaNet), which was collected from restaurant reviews on Yelp. Each review consists of one textual paragraph and multiple images. Entire dataset has already been split into a train, valid, and test set. According to the location of restaurants, a test set is divided into five subsets: Boston (BO), Chicago (CH), Los Angles (LA), New York (NY), and San Francisco (SF). The target label is the rating (from 1 to 5) of each review and it could be considered as a multi-classification problem. The statistics of them are shown in Table 1.

The number of images in each review is fixed to 3 and an additional global average image (MEAN) is added into each review. Truong et al. proposed the additional image has global visual information which could improve the robustness. Following the same preprocess, each textual content corresponds to four images. GloVe was employed for word embedding in VistaNet, while pre-trained BERT (base-uncased), the most popular language model in natural language processing, is utilized in our method to encode each word as a 768-dimensional vector. In the process of fine-tuning BERT, the number of words in each review is fixed to 256 and the batch size is set to 32. Transformers module [48] is exploited to fine-tuning BERT with a 2e−5 learning rate for 4 epochs. Following baselines are employed to compare with our proposed model on multimodal sentiment classification.

- TFN: It was firstly proposed by Zadeh et al. [39], which utilizes the outer product of different modal feature vectors as the fusion representation. Since there are multiple images for each review, the pooling layer is applied to aggregate visual information. Therefore, two variants are presented in the experiments, in which average pooling is employed in TFN-avg and max pooling is employed in TFN-max for all images before concatenating with text feature vectors.
- BiGRU: The classic model proposed by Tang et al. [49] could capture forward and backward dependence based on a bi-directional gated recurrent unit. Average pooling and max pooling are applied to yield two variants BiGRU-avg and BiGRU-max.
- HAN: Yang et al. [50] proposed the attention network for text classification, which could hierarchically extract the representation of words, sentences, and documents. Although HAN was proposed only for textual modality, it is utilized to generate textual representations that are concatenated with visual representations as the input of a downstream classifier. HAN-avg and HAN-max are two variants that correspond to average and max pooling.
- FastText: Bojanowski at al. [51] proposed to enrich the word representations with sub-word information. It has a simple network architecture, but has a competitive performances on text classification. It is employed to generate word embedding representations as a comparison with BERT.
- Glove: It is a popular language model applied in numerous text-related problems [52]. Global matrix factorization and local context window are employed to extract both global and local information from word sequences. It is also employed in VistaNet to obtain word representations.
- BERT: The pre-trained language model proposed by Devlin et al. [11] can capture very long-term dependence based on multi-head attention. The textual contents in a train set are employed to fine-tune BERT on sequential classification task.
- VistaNet: Truong et al. [10] employed visual feature as query and proposed visual aspect attention to fusion textual and visual features.

After fine-tuning BERT, it is utilized as an encoder for word vectors. Visual features extracted by pre-trained VGG are directly provided by the dataset. These features are employed without any other processing to ensure the fairness of experimental comparison with VistaNet. The Adam optimizer is applied in the training process and the learning rate is set to 2e−5. The batch size is 128 and the number of attention heads is 12. To prevent the over-fitting problem, the dropout rate is set to 0.6 and the parameter of weight decay in Adam is set to 10. The hyper parameter α for similarity loss is set to 0.1. Other hyper parameters of the experiments are listed in Table 2. The classification accuracy on five test sets are shown in Table 3. Notice that, the weighted average accuracy based on sample amounts of five test sets is shown in the last column as a comprehensive metric of generalization capability.

Two popular language models (FastText and Glove) are employed to compare with BERT, and their word representations are also utilized in HFN to evaluate our proposed fusion methods. The dimension of word representations in FastText and Glove is set to 300. The average pooling is conducted on word representations to obtain a sentence-level representation as the replacement of $T_{\mathrm{CLS}}$ in BERT. As shown in Table 3, HFN with max pooling and BERT has obtained the highest accuracy on three test sets except for CH and NY. According to the weighted average accuracy, HFN-max has the highest comprehensive accuracy and the best generalization ability. The pooling layer is employed to aggregate visual information of multiple images and max pooling is better than average pooling for most methods in Table 3, including TFN, BiGRU, and HAN. HFN is robust for the choice of pooling layer, which also demonstrates the generalization of our proposed model.

### 4.2. Comparative Experiments on CMU-MOSI and CMU-MOSEI Datasets

Two additional datasets, CMU-Multimodal Opinion Sentiment Intensity (CMU- MOSI) [53] and CMU-Multimodal Opinion Sentiment and Emotion Intensity (CMU- MOSEI) [54] are employed to evaluate and compare the performance of HFN. Each record in CMU-MOSI and CMU-MOSEI is a segment of a YouTube speech video, which is composed of textual, acoustic, and visual modalities. CMU-Multimodal SDK [55] is utilized to download, align, and split two datasets. The statistics of two datasets after preprocessing are shown in Table 4.

To satisfy the condition of single text and multiple images, acoustic signals are abandoned and only the first four images are exploited in each record. Visual features are directly provided by CMU-Multimodal SDK, which are extracted by FACET, including facial action units and face poses. The label of each record in CMU-MOSI and CMU-MOSEI is a real number between −3.0 and 3.0, representing the sentiment score. According to the scores, the regression problem is translated into two classification problems: 2-class (non-negative, negative) and 7-class (ranging from −3 to 3). Correspondingly, binary accuracy (Acc-2) and seven-class accuracy (Acc-7) are utilized as the metrics as shown in Table 5.

HFN with average pooling has achieved the highest accuracy for both the 2-class and 7-class classification task on CMU-MOSI. Although the performances of each method on the 2-class classification task for CMU-MOSEI are close, the HFN-avg has the best performance on seven-class accuracy. Both two datasets are collected from YouTube videos, and CMU-MOSEI is a extended version of CMU-MOSI. The essential difference between them and Yelp datasets is that images in CMU-MOSI and CMU-MOSEI could provide sufficient and complete sentiments and emotions. It is necessary to discover the bidirectional interaction between text and images, but HFN has obtained an effective classification performance with multi-head visual attention. In addition, training samples of CMU-MOSI are not enough which cause its classification task more difficulty than CMU-MOSEI, and the 7-class classification task is more complex than the 2-class classification task. According to the results of Table 5, it could be found that HFN has a better generalization capability for complex tasks.

### 4.3. Comparative Experiments on Twitter-15 and Twitter-17 Datasets

In order to further evaluate the adaptability of the proposed model on different platforms, the additional experiments are conducted on the datasets collected from Twitter. Twitter-15 and Twitter-17 are two datasets released for target-oriented multimodal sentiment classification [42], which meet the inductive bias of our method, i.e., the image within a text-image post or review only plays an augmentative role, which cannot deliver complete information on their own. Two datasets have already been split into train, valid, and test set. The basic statistics of Twitter-15 and Twitter-17 are shown in Table 6.

Note that, each record in two datasets has provided annotated target terms (i.e., entities or aspects) for the instruction of capturing the target-oriented sentiment information. Target terms are concatenated with the related context as the complete textual content in this experiment. The label of each record indicates three categories of sentiment polarity (i.e., negative, neutral, and positive). The number of attention heads is set to 8, and the hyper parameter α for similarity loss is set to 0.1. The learning rate is set to 2e−4, and the batch size is 16. Since each record in Twitter-15 and Twitter-17 is attached with only one image, the pooling layer of HFN, employed to aggregate the features from multiple images, is removed in this experiment.

Seven competitive approaches are employed to evaluate our model, in which MemNet [56] employs a multi-hop attention mechanism to capture the relevant information; RAM [57] applies a GRU module to update the queries for multi-hop attention mechanism; ESTR [24] extracts the relevant information from both a left and right context with the target query; MIMN [58] adopts a multi-hop memory network to model the interactive attention between the textual and visual context; ESAFN [24] is an improved version of ESTR with a visual gate to control the fusion of visual information; and TomBERT [42] is a multimodal fusion model based on a multi-head attention mechanism. The representation fusion approach of TomBERT and our proposed model is similar, but the textual and visual context are equally treated in TomBERT. Besides, the decision fusion is not considered in TomBERT.

According to the experimental results shown in Table 7, HFN has achieved the best performance on both Twitter-15 and Twitter-17 datasets. The performance of text-based methods is still relatively limited except for BERT. BERT has a competitive performance even compared with two multimodal methods (MIMN and ESAFN) that have sophisticated architectures. This suggests that the multi-head attention mechanism plays a crucial role in information extraction, and the textual content can provide relatively complete semantic and sentimental information for learning a discriminative representation. Besides, ESAFN and TomBERT are both proposed by Yu et al., and two models have similar architectures but different attention mechanisms. The dot-product and vanilla attention mechanism are employed in ESAFN, but the multi-head attention mechanism is employed in TomBERT. From the comparative results, it is clearly observed that the performance improvement by multi-head attention mechanism is significant. Compared with ESAFN and TomBERT, our proposed model also applies the multi-head attention mechanism, but explicitly assigns different importance to the textual and visual content. The results in Table 7 can prove the effectiveness of our views and approaches.

### 4.4. Ablation Analysis for Representation Fusion

In the intermediate fusion stage, a multi-head visual attention is proposed for representation fusion and this process can be split into three steps: (1) The fusion of word vectors by visual attention; (2) the fusion of multiple images by self-attention; and (3) the fusion of high level representation by element-wise additive. The ensemble decision part of HFN is fixed and the ablation experiments are conducted to evaluate the impacts of three representation fusion steps. Besides, the fusion performances of HFN are also compared with common fusion methods: Element-wise additive (Add), element-wise multiply (Mul), and concatenating (Concat), in which $T_{\mathrm{CLS}}$ and the average of *G* are utilized as textual and visual representation. As shown in Table 8, each step in multi-head visual attention has an improvement over the previous fusion which proves three fusion steps are efficient and necessary. The comparison results with other fusion methods also prove the effectiveness of the multi-head visual attention.

Note that NY has shown a different trend compared with the other cities in Table 8. The third fusion step has not achieved the expected improvement with the element-wise additive of textual representation $T_{\mathrm{CLS}}$ and fusion representation *F*. It is caused by the inaccurate information provided by either $T_{\mathrm{CLS}}$ or *F*. However according to the result of Add on the NY test set, textual representation has captured accurate sentiment information. Therefore, the different trend is caused by fusion representation, which relies on the match of visual and textual contents. For visual-textual sentiment analysis, it assumes that there is a implicit correlation between visual and textual contents. When the realistic problem does not meet the assumption, the unimodal method could even be better than multimodal fusion methods.

### 4.5. Visualization for Decision Fusion and Diversity

**Figure 5:**
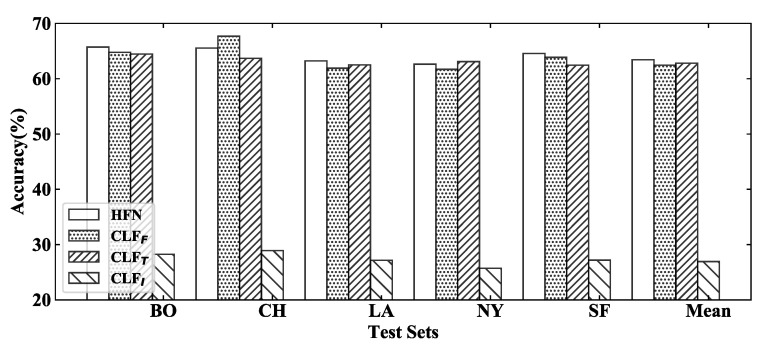
Individual classifiers are expected to output decisions close to true labels which is significant for subsequent decision fusion.

Individual classifiers are trained to make independent decisions with textual, visual, and fusion representations. Decision fusion based on the attention mechanism is employed to make the final decision with the decision supports from individual classifiers. In addition, decision diversity is injected into the whole model via a similarity penalty. In the training process, individual classifiers are expected to be a real classification module which could make a decision close to true labels, rather than a feature extractor. As shown in Figure 5, individual classifiers could also have high accuracy which are conductive to a decision fusion stage. Obviously, ${\mathrm{CLF}}_{I}$ with visual features has the worst performance, and this phenomenon could prove our view that visual features in reviews cannot tell a complete story, but only play an augmentative role for textual information.

**Figure 6:**
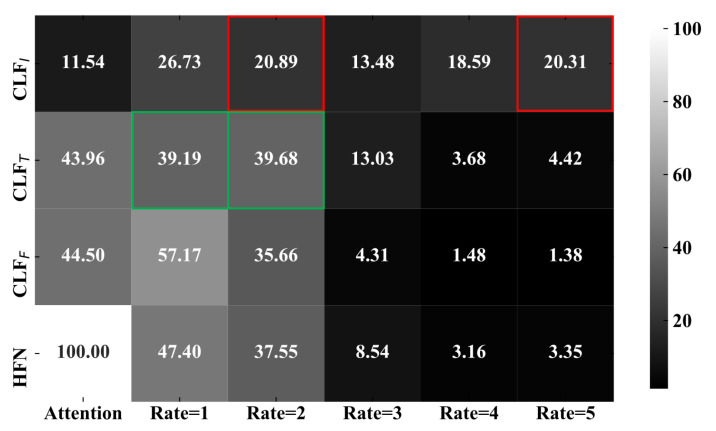
A neural network is trained to measure the importance of classifiers and the final decision is determined by adaptive attention weighted based on prediction results of ${\mathrm{CLF}}_{F}$, ${\mathrm{CLF}}_{T}$, and ${\mathrm{CLF}}_{I}$. Decision diversity has guaranteed the generalization and robustness of the whole model.

The decision fusion method is proposed to ensemble prediction results from each base classifier and make the final decision, in which a neural network is trained to measure the importance of classifiers. Figure 6 is employed to visualize the decision fusion process and adaptive attention scores. Each row corresponds to the decisions of a classifier and the bottom row denotes the final predictions. The scores of sixth columns (ranged from Rate = 1 to Rate = 5), represent the probability distribution of each classifier whose values accumulative total in each row is 100. The first column denotes the attention distribution generated by the neural network in the decision fusion process. The attention scores are assigned to three base classifiers (${\mathrm{CLF}}_{I}$, ${\mathrm{CLF}}_{T}$, and ${\mathrm{CLF}}_{F}$), and the weighted sum is the final decision of HFN.

In the decision fusion process, each base classifier firstly predicts the probability of target labels and is enforced to make diverse decisions by a similarity penalty. Then, different adaptive attention scores (the first column) are assigned to the classifiers. At last, the final decision (the bottom row) is determined based on the attention weighted. As shown in Figure 6, ${\mathrm{CLF}}_{T}$ has assigned similar probability to Rate = 1 and Rate = 2, because it is difficult to distinguish them only using single text modality. The same problem has also accrued in ${\mathrm{CLF}}_{I}$ that Rate = 2 and Rate = 5 have similar visual information. Therefore, benefiting from the decision diversity, the decision fusion has improved both the accuracy and generalization of the whole model based on the complementary effect classifiers.

### 4.6. Analysis on Hyper Parameter

**Figure 7:**
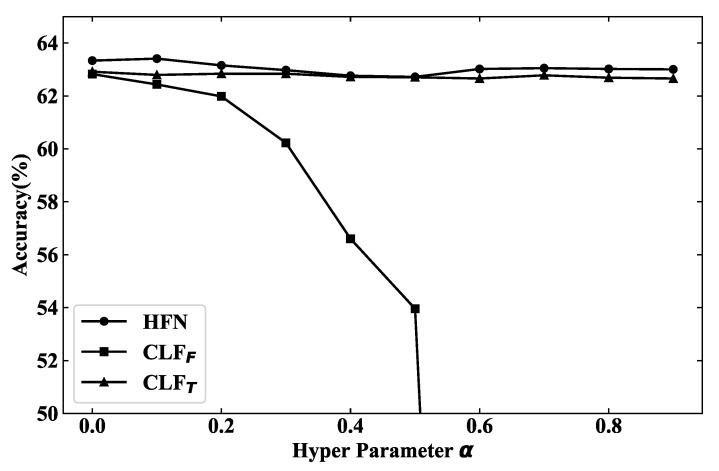
Benefiting from decision diversity, the hybrid neural network keeps stable results although the accuracy of ${\mathrm{CLF}}_{F}$ drops sharply with the increasing of α.

The hyper parameter α is utilized to balance the decision and similarity loss which are proposed to improve both the accuracy and diversity of prediction results from independent classifiers. The value of α is changed from 0 to 1 with a step of 0.1 and the results of each classifier are shown in Figure 7. Since the accuracy of ${\mathrm{CLF}}_{I}$ fluctuates between 26.90% and 27.30%, it is not shown in the figure for better visualization.

It is obvious that the accuracy of ${\mathrm{CLF}}_{F}$ drops sharply with the increasing of α, while the results of ${\mathrm{CLF}}_{T}$ are stable only using textual features. The reason is that the whole model will focus on the decision diversity of classifiers instead of classification accuracy when α is too large. Since the architecture of ${\mathrm{CLF}}_{T}$ is much simpler (only one fully connected layer) than that of ${\mathrm{CLF}}_{F}$, ${\mathrm{CLF}}_{T}$ could converge earlier which will make ${\mathrm{CLF}}_{F}$ harder to converge because of the cosine similarity.

HFN has reached the highest accuracy at α=0.1, and it could keep good results with the changing of α, even the accuracy of ${\mathrm{CLF}}_{F}$ continues to drop. The results illustrate that the hybrid fusion network could benefit from decision diversity and multiple classifiers are better than a single one. The accuracy of the hybrid fusion network is always higher than ${\mathrm{CLF}}_{T}$ which also prove the decision fusion is not simply forwarding the decision of ${\mathrm{CLF}}_{T}$, but is adaptively learning the importance or confidence of each classifier and making the final prediction with decision diversity.

## 5. Conclusions

A hybrid fusion network is proposed for multimodal sentiment classification in an online social network. It captures both the common inter-modal and unique intra-modal information based on the intermediate representation fusion and the late decision fusion. In the intermediate stage, multiple images are exploited as queries to extract principal information from textual content based on the multi-head visual attention. To improve the generalization and capture the intra-modal characteristics, a decision fusion method is proposed to make the final decision based on diverse decision supports from individual classifiers. The cosine similarity is added into the loss function to promote the decision diversity between classifiers. Empiric results on known multimodal datasets have shown that our hybrid fusion network could achieve a higher accuracy and better generalization for sentiment classification.

However, there are still some limitations of the proposed model. Firstly, the quality and quantity of training samples have limited the performances. Secondly, the classification accuracy and decision diversity are two conflict objectives when the model is about to converge. In future work, we plan to build multimodal corpus and find more effective methods to balance two training objectives. Besides, our work concerns global sentiment information, rather than fine-grained local sentiments. Aspect-level multimodal sentiment analysis is our next research direction, which is a more complex problem and requires more accurate semantic representations.

## Figures and Tables

**Table 1:** Statistics of the Yelp dataset.

Datasets	#Docs	Avg. #Words	Max. #Words	Min. #Words	Avg. #Images	Max. #Images	Min. #Images
Train	35,435	225	1134	10	5.54	147	3
Valid	2215	226	1145	12	5.35	38	3
BO	315	211	1099	14	5.25	42	3
CH	325	208	1095	15	5.60	97	3
LA	3730	223	1103	12	5.43	128	3
NY	1715	219	1080	14	5.52	222	3
SF	570	244	1116	10	5.69	74	3

**Table 2:** Settings of the hyper parameters.

Hyper Parameters	Settings
optimizer type	Adam
learning rate	2e−5
weight decay	10
batch size	128
dropout rate	0.6
the amount of attention heads	12
the weight of similarity loss α	0.1
the dimension of visual representation	4096
the dimension of textual representation	768
the amount of words in each review	256
the amount of images attached to each review	4

**Table 3:** Performance comparison to baselines on classification accuracy.

Methods	BO	CH	LA	NY	SF	Mean
TFN-avg	46.35	43.69	43.91	43.79	42.81	43.89
TFN-max	48.25	47.08	46.70	46.71	47.54	46.87
BiGRU-avg	51.23	51.33	48.99	49.55	48.60	49.32
BiGRU-max	53.92	53.51	52.09	52.14	51.36	52.20
HAN-avg	55.18	54.88	53.11	52.96	51.98	53.16
HAN-max	56.77	57.02	55.06	54.66	53.69	55.01
FastText	61.27	59.38	55.49	56.15	55.44	56.12
Glove	60.00	59.38	55.76	55.86	56.14	56.20
BERT	62.13	62.33	60.79	60.51	61.86	60.95
VistaNet	63.81	65.74	62.01	61.08	60.14	61.88
HFN-avg (FastText)	65.40	**68.00**	62.36	61.69	62.81	62.64
HFN-max (FastText)	**65.71**	66.15	62.95	62.39	60.35	62.87
HFN-avg (Glove)	64.76	66.46	62.39	62.68	63.86	62.90
HFN-max (Glove)	**65.71**	65.84	62.84	**63.15**	61.75	63.11
HFN-avg (BERT)	**65.71**	65.54	63.06	62.97	64.21	63.38
HFN-max (BERT)	**65.71**	65.54	**63.22**	62.62	**64.56**	**63.41**

**Table 4:** Statistics of CMU-MOSI and CMU-MOSEI.

Statistics	CMU-MOSI	CMU-MOSEI
#Train	1283	16,315
#Valid	229	1871
#Test	686	4654
#Textual Features	768	768
#Visual Features	47	35
Length of Sequences	20	30

**Table 5:** Performance comparison over CMU-MOSI and CMU-MOSEI.

Methods	CMU-MOSI			CMU-MOSEI		
	Acc-2	F1	Acc-7	Acc-2	F1	Acc-7
TFN-max	71.14	71.26	27.55	82.53	82.41	49.38
TFN-avg	69.53	69.80	30.47	82.06	82.15	48.89
BiGRU-max	72.16	72.33	32.80	**82.83**	83.77	50.86
BiGRU-avg	72.59	72.75	33.67	82.75	83.63	50.58
HFN-max	73.03	73.46	34.26	82.61	**83.92**	51.20
HFN-avg	**74.49**	**75.07**	**35.42**	82.36	83.60	**51.65**

**Table 6:** Statistics of Twitter-15 and Twitter-17.

Datasets	Twitter-15				Twitter-17			
	#Docs	Avg. #Words	Max. #Words	Min. #Words	#Docs	Avg. #Words	Max. #Words	Min. #Words
Train	3179	16.72	35	2	3562	16.21	39	5
Valid	1122	16.74	40	2	1176	16.37	31	6
Test	1037	17.05	37	2	1234	16.38	38	6

**Table 7:** Performance comparison over Twitter-15 and Twitter-17.

Methods	Twitter-15		Twitter-17	
	Acc	Macor-F1	Acc	Macor-F1
MemNet	70.11	61.76	64.18	60.90
RAM	70.68	63.05	64.42	61.01
BERT	74.15	68.86	68.15	65.23
ESTR	71.36	64.28	65.80	62.00
MIMN	71.84	65.69	65.88	62.99
ESAFN	73.38	63.98	66.13	63.63
TomBERT	76.37	72.60	69.61	67.48
HFN	**78.62**	**73.83**	**71.35**	**68.52**

**Table 8:** Performance comparison of the representation fusion methods on classification accuracy.

Methods	BO	CH	LA	NY	SF	Mean
Concat	63.81	63.38	61.80	61.69	63.16	62.06
Add	64.76	62.15	62.52	**63.44**	61.40	62.75
Mul	65.40	63.38	62.65	62.68	63.16	62.87
Step1	64.13	64.62	61.64	61.98	63.51	62.15
Step1 + 2	64.76	64.92	62.92	**63.44**	63.16	63.26
Step1 + 2 + 3	**65.71**	**65.54**	**63.22**	62.62	**64.56**	**63.41**

## Data Availability

The datasets involved in this study are available in publicly accessible repositories. Yelp dataset can be found in: https://github.com/PreferredAI/vista-net. CMU-MOSI and CMU-MOSEI datasets are published in: https://github.com/A2Zadeh/CMU-MultimodalSDK. Twitter-15 and Twitter-17 datasets can be found in: https://github.com/jefferyYu/TomBERT.
